# Key clinical and demographic factors influencing head and neck tumor severity in Lagos

**DOI:** 10.1186/s12903-025-07235-0

**Published:** 2025-12-01

**Authors:** Emmanuel Temitope Aladenika, Warith Olaitan Akinshipo, Adegbayi Adeola Adekunle, Tosin Bakare, Tamara Busch, Olajumoke Ajibola Effiom, Wasiu Adeyemo, Azeez Butali

**Affiliations:** 1 Iowa Institute for Oral Health Research, University of Iowa, Iowa City, IA USA; 2 Department of Oral Pathology, Radiology and Medicine, College of Dentistry, University of Iowa, Iowa City, IA USA; 3 Department of Oral and Maxillofacial Pathology/Biology, University of Lagos, Lagos, Nigeria; 4 Department of Oral and Maxillofacial Surgery, University of Lagos, Lagos, Nigeria

**Keywords:** Orofacial tumors, Head and neck neoplasm, Tumor severity prediction

## Abstract

**Background:**

Head and neck tumors pose a major health challenge in Nigeria, yet the factors that influence their severity remain poorly understood. No predictive model currently exists to assess malignancy risk in this population. This study aimed to identify key clinical and demographic predictors and to develop a model for malignancy risk in tumors diagnosed in Lagos, Nigeria.

**Methods:**

We analyzed patient records with tumor diagnoses from 2000 to 2024, obtained from the Oral and Maxillofacial Surgery and Oral Pathology departments of the Lagos University Teaching Hospital. Surgical biopsy records and corresponding histopathology reports were reviewed. Tumor sites were grouped into five anatomical regions: mandible, midface, oral mucosa and salivary glands, extra oral, and unspecified regions. Descriptive statistics summarized patient and tumor features, while statistical analyses examined associations between demographics and tumor severity (benign/malignant). A predictive model for tumor severity was developed using data-splitting and leave-one-out cross-validation approaches.

**Results:**

Of the 1,068 patients, 1,062 had confirmed diagnoses. Median age was 35 years [IQR:23–52], 53.96% were male, and the mandible was the most common site (48.31%). Tumors were most frequent on the right (30.13%) and measured 6 × 4x2cm on average. Median duration of presentation was 12 months [IQR:5–36], with 50.75% benign tumors. Tumor severity was significantly associated with age (47 vs. 28 years), site, laterality, duration (*p* < 0.001), and sex (*p* = 0.024), with males more susceptible to malignancy. Age inversely correlated with presentation duration (*p* = 0.00067), and tumor site was not associated with sex (*p* = 0.054). Conventional ameloblastoma was the most common benign tumor, while squamous cell carcinoma was the most common malignancy. Multivariate analysis showed malignancy was associated with age (OR = 1.61; *p* < 0.001), tumor site (midface (OR = 3.88), oral mucosa and salivary glands (OR = 23.8), and extraoral sites (OR = 108.01) compared to the mandible [all *p* < 0.001]), length (OR = 1.93; *p* < 0.001), and shorter duration (OR = 0.68; *p* < 0.001). Central (OR = 0.2), left (OR = 0.46), or right laterality (OR = 0.32) lowered malignancy odds versus unspecified (all *p* < 0.04). Our predictive model approaches achieved area under the curve of 0.864 and 0.883.

**Conclusions:**

We identified age, site, laterality, and duration before presentation, as key malignancy predictors. This will aid comprehensive care plans for optimal clinical outcomes.

**Supplementary Information:**

The online version contains supplementary material available at 10.1186/s12903-025-07235-0.

## Background

The orofacial region is a very important region involved in many daily physiologic activities like breathing, chewing and swallowing amongst others [1, 2]. As with other body parts, this region contains cells that function cooperatively to maintain these activities and homeostasis [3, 4]. However, some of the cells in orofacial region can proliferate abnormally and lead to different neoplastic conditions generally referred to as tumors [3, 5]. Tumors are typically classified mainly into benign and malignant tumors based on their clinical, biological, and histological characteristics [3, 4]. Benign tumors closely resemble normal, differentiated cells but may carry mutations that alter their growth and function without causing tissue invasion [3]. Conversely, malignant tumors invade surrounding tissues and may metastasize to distant organs, making them more life-threatening [3].

The exact etiopathogenesis of most tumors in the orofacial region remains widely unknown. However, genetic predisposition has been suggested, while environmental factors such as viral infection, dietary deficiencies, trauma, and alcohol and tobacco intake have been implicated as well [6]. Because of their location, orofacial tumors can cause disfigurement, disability, functional impairment, bone expansion, tooth mobility, and destruction of adjacent structures ultimately affecting the quality of life of individuals [7].

Tumors affecting the lower face are relatively common [5]. The lower face encompasses the entire mandible, supported by the overlying soft tissue, and includes the tongue [5]. Malignant lesions typically found in the lower face include sarcomas of soft and hard connective tissues, carcinomas of the salivary glands, with squamous cell carcinoma (SCC) accounting for over 90% of reported malignant tumors in the oral cavity [5]. Some cancers in this region are metastases from distant sites such as the breast, lungs, abdominal organs, or prostate gland [5]. Benign lesions in the lower face are often odontogenic or non-odontogenic tumors, predominantly ameloblastoma [5]. The distribution of orofacial tumors exhibits geographic variation which may be influenced by social, cultural, occupational, and climatic factors [7]. This is evidenced by the varying findings from Africa [8–15], Asia [16–18], North America [19], South America [20–22] and Europe [20, 23].

In Nigeria, several studies have shown the distribution and frequency of orofacial tumors across different parts of the country with some findings agreeing with each other and some others contrasting each other [6, 9, 12, 24–27]. Similarly, some studies have also explored association between tumor severity (benign or malignant) and patient characteristics such as sex, age and duration before presenting to the clinic [6, 9, 15, 24, 25].However, there are very few studies on the association between tumor characteristics and tumor severity in Nigeria. With most focusing on the tumor site and not exploring others like tumor laterality and tumor dimension [6]. Treatment of orofacial tumors varies according to the severity of the tumor [7]. Benign tumors are typically treated by surgical removal, while malignant lesions may require chemotherapy, surgical removal, or a combination of these methods [7]. However, in Africa, particularly Sub-Saharan Africa, orofacial tumors pose a significant health challenge due to patients who often present with advanced disease stages [4]. This delay in presentation is concerning, as it complicates treatment outcomes. Histopathology, which is essential for a definitive diagnosis, is typically performed at tertiary institutions. However, these institutions are often overwhelmed by the high volume of patients, as many bypass primary healthcare centers and self-refer directly to secondary or tertiary hospitals [28]. This presents a challenge, as healthcare personnel at these institutions must prioritize cases efficiently to manage the patient load.

Given the challenges identified in the management of tumors, it is essential to have a predictive model that can help prioritize patients based on tumor severity, starting at the secondary and extending to the tertiary health institutional levels. Such a model would streamline the referral process and ensure timely and appropriate care for patients with orofacial tumors. To the best of our knowledge, no predictive model currently exists in Nigeria to support clinical decision-making for head and neck tumors. Therefore, it is crucial to develop a model that can identify and prioritize patients based on the severity of their tumors. Hence, the aim of the current study was to explore demographic and clinical factors associated with head and neck tumor severity and to develop a preliminary predictive model that may support clinical triage of head and neck tumors in Lagos, Nigeria.

## Materials

### Study population and design

Our study was a cross-sectional, institution-based, retrospective study that examined biopsy records of patients diagnosed with head and neck tumors at a tertiary institution in Lagos, Nigeria (Lagos University Teaching Hospital, Idi araba, Lagos). Data were retrieved from departmental records, including histopathology forms and files maintained by the Department of Oral and Maxillofacial Surgery, as these contained the patient records for biopsies that were subsequently sent to the Oral Pathology laboratory for histopathological reporting. The review included records spanning the years 2000 to 2024.

### Ethical approval

Ethical approvals were obtained at the local institution review boards (IRBs): Health Research Ethics Committee (HREC) of Lagos University Teaching Hospital (LUTH) (ADM/DSCST/HREC/APP/7496) and Institutional Review Board at the University of Iowa (HawkIRB) (IRB ID #: 202,503,188).

### Study variables

The dataset included demographic and clinical information for each case, including age (in years), sex, year of diagnosis, tumor site, tumor laterality, histologic diagnosis site, pre-operative tumor dimensions (length, breadth, and height in centimeters), duration before presentation at the clinic, and histopathological diagnosis. To improve model performance in downstream analyses, tumor sites were consolidated into five anatomical categories based on proximity and clinical relevance: mandible, midface, oral mucosa & salivary glands, extraoral, and unspecified. Tumors located in the maxilla, hard/soft palate, cheek, antrum, zygoma, and periauricular region were grouped under midface, representing structures within or adjacent to the midfacial skeleton. Sites such as the gingivae, tongue, floor of the mouth, buccal and labial mucosa, lips, parotid gland, sublingual gland, and submandibular gland were categorized under oral mucosa & salivary glands, encompassing mucosal and major salivary gland sites within the oral cavity proper. Tumors in visibly external regions, including the forehead, nose, neck, submandibular region (involving overlying skin), and submental region, were classified as extraoral. Tumors with both intraoral and extraoral components were categorized based on the location most prominently reported in the patient records. Cases with tumors involving multiple locations or lacking specific site data were categorized as unspecified.

Tumor laterality was categorized into five groups: right, left, midline, right-anterior-left, and unspecified. Histological diagnoses were further classified into two categories based on severity: benign and malignant.

### Statistical analysis

#### Univariate

The distribution of continuous variables was initially assessed graphically for normality. Descriptive statistics for continuous variables were then summarized using the median and interquartile range (IQR) to present participants’ sociodemographic details and tumor characteristics. Categorical variables were described using frequencies and percentages.

#### Bivariate

Chi-square test was used to assess associations between tumor severity and categorical variables, such as sex, tumor site, and tumor laterality, as its assumptions were not violated. For continuous variables, which were not normally distributed, the Mann–Whitney U test was employed to assess associations between tumor severity and variables such as age, duration before presentation to the clinic, and tumor dimensions (length, breadth, and height). Spearman’s rank correlation coefficient (rs) was used to evaluate the strength of the association between age and duration before presentation, given that both variables were non-normally distributed. Spearman’s rank correlation coefficient was interpreted as follows: 0.10 = weak, 0.30 = moderate, and 0.50 = strong. Additionally, Mann–Whitney U test was conducted to examine the association between sex and duration before presentation, as well as Chi-square tests for association between tumor site and sex.

#### Multivariate

Non-normally distributed continuous variables were first transformed using a square root transformation to facilitate better model performance. Square root transformation was chosen due to its ability to stabilize variance. A logistic regression model was then used to evaluate variables associated with tumor severity, including all potential predictors. For tumor site, the mandible was selected as the reference category because it was the most frequently reported location in both our dataset and the literature. For tumor laterality, the unspecified category was used as the reference. This category likely reflects cases with incomplete diagnostic documentation and provides a neutral baseline, allowing us to assess how clearly defined laterality compared to cases where laterality was not reported. All variables, including transformed continuous ones, were initially included in the model. Multicollinearity was then assessed using the generalized variance inflation factor (GVIF) from the “car” R package, where values above 4 indicate concern and those exceeding 10 require correction. Tumor length, breadth and height had GVIF values over 4, indicating multicollinearity. Tumor breadth and height were subsequently excluded. The model was then reassessed, ensuring acceptable GVIF values for the remaining variables. The logistic regression equation for the final model is:

$$\begin{aligned}\mathrm{Log}({\mathrm p}_{\mathrm i}/1-{\mathrm p}_{\mathrm i})&={\mathrm\beta}_0+{\mathrm\beta}_1\surd{\mathrm{Age}}_{\mathrm i}+{\mathrm\beta}_2\mathrm I({\mathrm{Sex}}_{\mathrm i})\\&+\;{\mathrm\Sigma}_{\mathrm j}\;{\mathrm\beta}_{3\mathrm j}\mathrm I({\mathrm{Site}}_{\mathrm i}=\mathrm j)\\&+{\mathrm\Sigma}_{\mathrm k}\;{\mathrm\beta}_{4\mathrm k}\mathrm I({\mathrm{Laterality}}_{\mathrm i}=\mathrm k)\\&+{\mathrm\beta}_5\surd{\mathrm{length}}_{\mathrm i}+{\mathrm\beta}_6\surd\mathrm{Duration_i}\end{aligned}$$

To build the final predictive model, observations with missing data were excluded, hence, retaining 916 complete cases. To ensure rigor, 2 approaches were used.

1. Data splitting approach: The dataset was randomly divided into training and validation subsets, using a fixed random seed for reproducibility.
2. Leave-one-out cross-validation (LOOCV) approach: Each observation in the dataset was treated as a single validation case, while the remaining data served as the training set.

For the data-splitting approach, predictor variables included those that were significantly associated with tumor severity in the bivariate analysis (*p* < 0.05): age, sex, tumor site, laterality, and duration before presenting to the clinic. An iterative selection process was used, where variables were removed one at a time, and models were compared based on the Akaike information criterion (AIC). Initially, the model excluding sex had the lowest AIC (Table 1).

**Table 1:** AIC for predictive variables in the full model. The model without sex was chosen since it has the lowest AIC

Variables in model	AIC
Age + Sex + Site + Laterality + Duration	627.98
Sex + Site + Laterality + Duration	692.69
Age + Site + Laterality + Duration	626.23
Age + Sex + Laterality + Duration	719.41
Age + Sex + Site + Duration	634.72
Age + Sex + Site + Laterality	743.19

Another round of iterative selection was conducted, further removing variables one at a time from this model. The refined model, which and had the lowest AIC (Table 2), was finally selected and validated.

**Table 2:** AIC for predictive variables in the model without sex

Variables in model	AIC
Age + Site + Laterality + Duration	790.02
Site + Laterality + Duration	864.1
Age + Laterality + Duration	918.36
Age + Site + Duration	797.81
Age + Site + Laterality	910.76

The logistic regression equation for the final model was:

1 $$\begin{aligned}\mathrm{Log}({\mathrm p}_{\mathrm i}/1-{\mathrm p}_{\mathrm i})&={\mathrm\beta}_0+{\mathrm\beta}_1\surd{\mathrm{Age}}_{\mathrm i}\\&+{\mathrm\Sigma}_{\mathrm j}\mathrm\beta{}_{3\mathrm j}\mathrm I({\mathrm{Site}}_{\mathrm i}=\mathrm j)\\&+{\mathrm\Sigma}_{\mathrm k}{\mathrm\beta}_{4\mathrm k}\mathrm I({\mathrm{Laterality}}_{\mathrm i}=\mathrm k)\\&+{\mathrm\beta}_5\surd{\mathrm{Duration}}_{\mathrm i}\end{aligned}$$

For the LOOCV approach, cross-validation was conducted both manually and using the caret package in R to enhance model reliability.

Model performance was assessed using the receiver operating characteristic (ROC) curve and the area under the curve (AUC) to measure discriminative ability. Sensitivity and specificity were also calculated to evaluate predictive accuracy. A 0.5 probability threshold was used, classifying tumors as malignant if *p* > 0.5 and benign otherwise.

#### Trend

The trend in the number of tumor diagnoses was also analyzed, including both malignant and benign cases, over the years from 2000 to 2024.

All analyses were conducted using R version 4.4.1. Statistical significance was assessed with a threshold of *P* < 0.05.

## Results

### Univariate analysis

Of 1,068 patients with tumors, 1,062 received definitive diagnoses. Table 3 summarizes patients’ demographics and tumor characteristics.

**Table 3:** Demographic and Tumor Characteristics of Participants (*N* = 1,062)

Variables	Total n (%)
Age in years (Median [IQR])	35 [23–52] years
Sex	
Male	573 (53.96%)
Female	489 (46.04%)
Site of tumor	
Mandible	513 (48.31%)
Midface	319 (30.04%)
Oral mucosa and salivary glands	154 (14.5%)
Extra oral	44 (4.14%)
Unspecific	32 (3.01%)
Laterality of tumor	
Right	320 (30.13%)
Left	311 (29.28%)
Central	32 (3.01%)
From right to left	174 (16.38%)
Unspecified	225 (21.19%)
Length in cm (Median [IQR])	6 [3–10] cm
Breadth in cm (Median [IQR])	4 [2–7] cm
Height in cm (Median [IQR])	2 [0.8–5] cm
Duration before presenting in months (Median [IQR])	12 [5–36] months
Severity	
Malignant	523 (49.25%)
Benign	539 (50.75%)

**Fig. 1:**
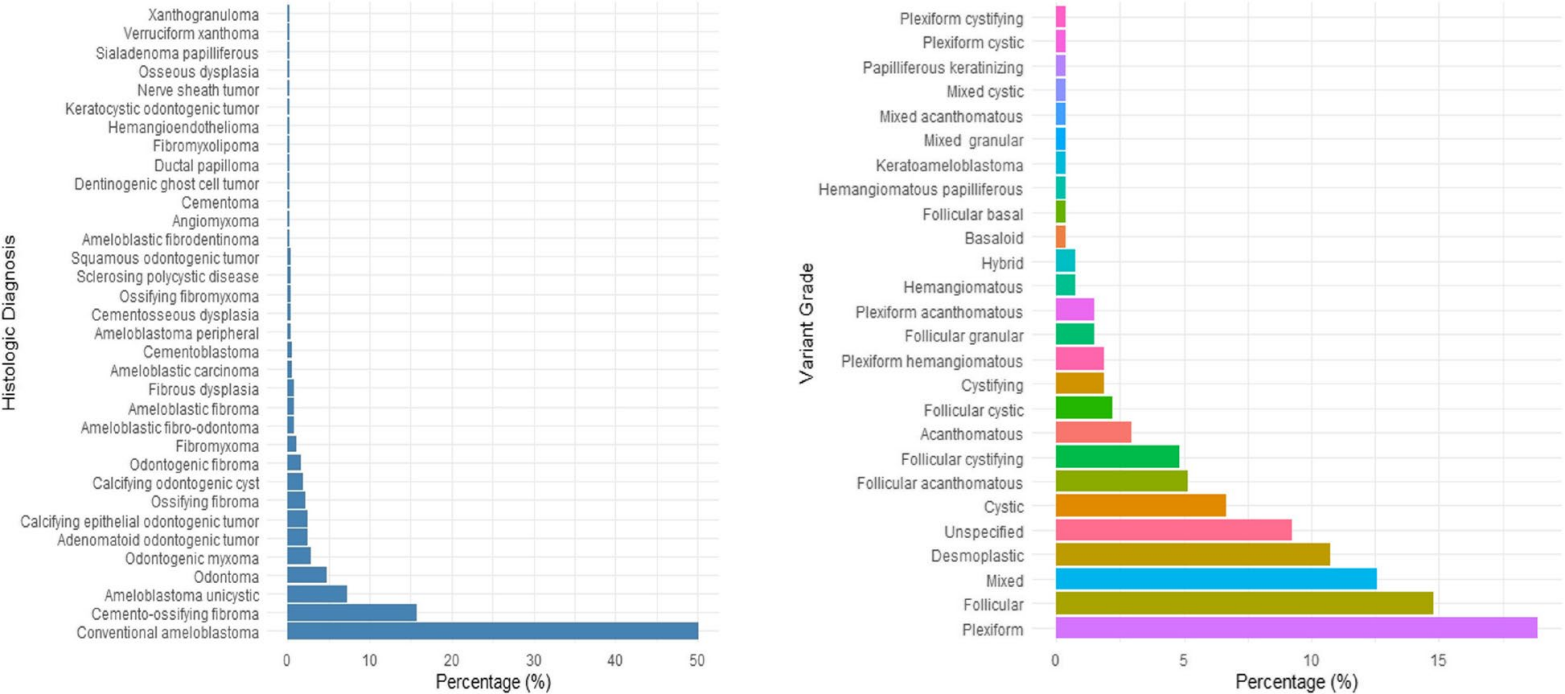
Distribution of histologic diagnoses and tumor differentiation in benign cases. Left Panel: Distribution of various histologic diagnoses among patients with benign head and neck tumors. Right Panel: Distribution of variants observed in conventional ameloblastoma cases

**Fig. 2:**
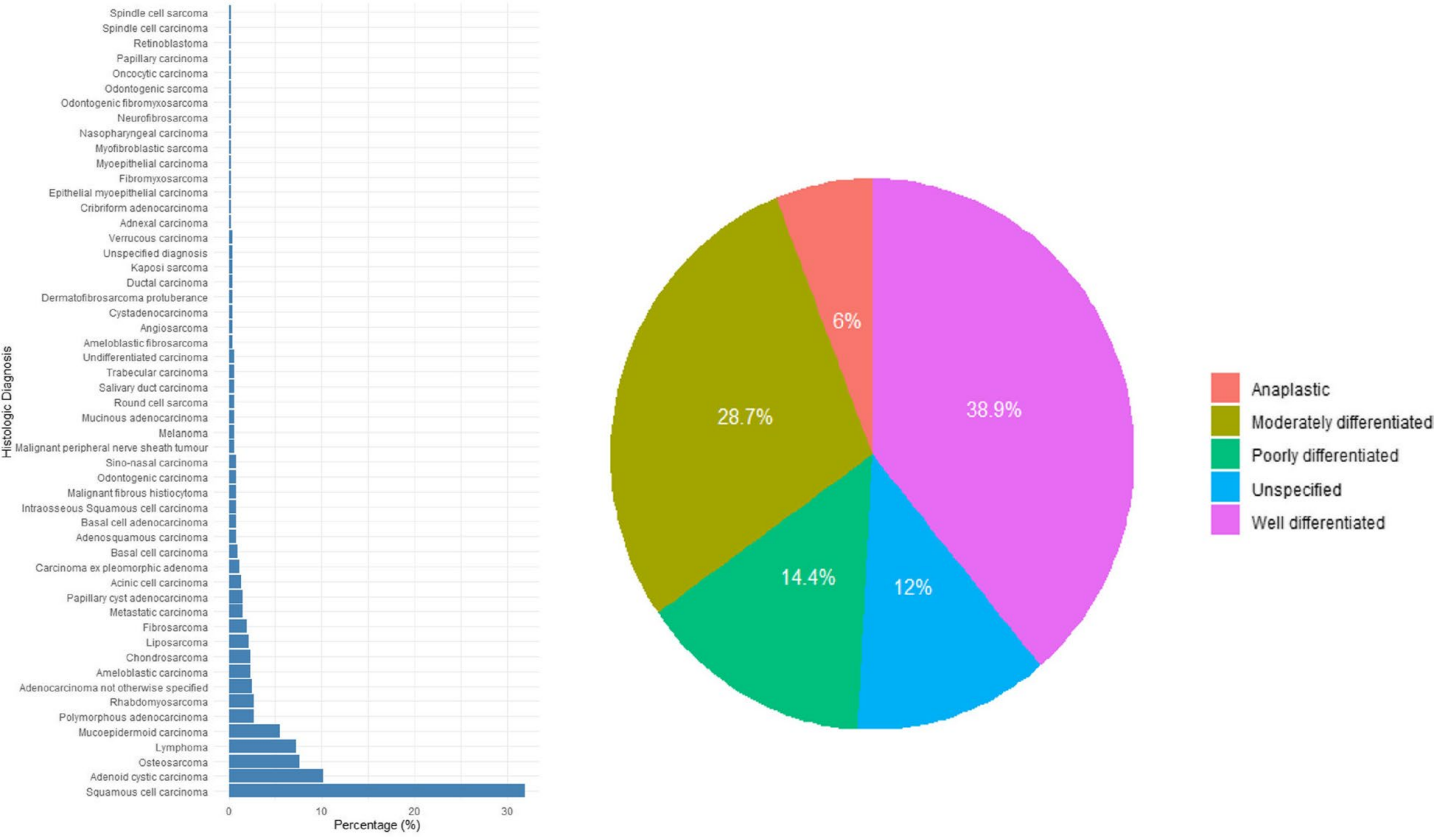
Distribution of histologic diagnoses and tumor differentiation in malignant cases. Left panel: Distribution of various histologic diagnoses among patients with malignant head and neck tumors. Right panel: Distribution of differentiation grades of squamous cell carcinoma

As shown in Table 1, the study population was relatively young, with the median age in the mid-thirties and a nearly equal sex distribution. The mandible was the most common tumor site, followed by midface and oral mucosa and salivary glands. Laterality was fairly balanced, though right-sided tumors were slightly more frequent. About half of all tumors were benign, most commonly conventional ameloblastoma, which showed a strong predilection for the mandible. Conventional ameloblastoma had a slight male predominance (M: F = 1.16:1), with the plexiform variant being the most frequent subtype (Fig. 1). Malignant tumors made up the remaining tumor cases, with squamous cell carcinoma (SCC) as the leading type. SCC showed a stronger male predominance (M: F = 1.83:1), with the well-differentiated variant being most common (Fig. 2).

### Bivariate analysis

Bivariate analysis identified significant associations between tumor severity and age, sex, site, laterality, and symptom duration (Table 4).

**Table 4:** Association between patient demographics, tumor characteristics, and tumor severity

**Characteristics**	**Malignant/Benign**	
	**Malignant (%)**	***P* value**
Overall sample	523 (49.25%)	
Age		< 0.001^$^
Sex		0.024^*^
Male	301 (52.53%)	
Female	222 (45.4%)	
Site		< 0.001^*^
Mandible	126 (24.56%)	
Midface	194 (60.82%)	
Oral mucosa and salivary glands	141 (91.56%)	
Extra oral	43 (97.73%)	
Unspecific	19 (59.38%)	
Laterality		< 0.001^*^
Right	152 (47.5%)	
Left	160 (51.45%)	
Central	10 (31.25%)	
From right to left	36 (20.69%)	
Unspecified	165 (73.33%)	
Duration		< 0.001^$^
Length		0.4318^$^
Breadth		0.6803^$^
Height		0.375^$^
	Duration	
Sex		0.051^$^
Age		0.00067^#^ (corr = −0.112)
	Male (%)	
Site		0.054^*^
Mandible	261 (50.88%)	
Midface	186 (58.31%)	
Oral mucosa and salivary glands	77 (50%)	
Extra oral	30 (68.18%)	
Unspecific	19 (59.38%)	

Additionally, associations between demographics and duration before presentation, and between tumor site and gender

^*^Chi square test

^$^Mann–Whitney U test

^#^Spearman correlation

As shown in Table 4, tumor severity was significantly associated with age, sex, site, laterality, and duration before presentation. Older patients were more likely to present with malignant tumors (median age: 47 years for malignant tumors vs. 28 years for benign, *p* < 0.001). Men had slightly higher rates of malignancy than women (*p* = 0.024). Tumor site was strongly associated with malignancy (*p* < 0.001), with the highest rates in the extraoral region. Laterality was also linked to tumor severity (*p* < 0.001), with unspecified tumors having the highest malignancy rate and central tumors having the lowest. A shorter duration of symptoms was associated with an increased odd of malignancy (*p* < 0.001). Tumor size (length, breadth, height) was not associated with malignancy (*p* > 0.3). Sex did not influence symptom duration (*p* = 0.051); however, age and duration were negatively correlated (Spearman’s ρ = −0.112, *p* = 0.00067), with older patients presenting sooner. Tumor site was not associated with sex (*p* = 0.054).

### Multivariable analysis

Multivariable logistic regression identified key factors associated with tumor malignancy (Table 5).

**Table 5:** Logistic regression model of demographic variables and tumor characteristics associated with tumor severity

Variables	OR	95% CI	*P* value
Age	1.61	1.41–1.84	< 0.001
Sex			
Female	Baseline	Baseline	Baseline
Male	1.1	0.73–1.66	0.66
Site			
Mandible	Baseline	Baseline	Baseline
Extra oral	108.01	17.12–2156.94	< 0.001
Midface	3.88	2.46–6.18	< 0.001
Oral mucosa and salivary glands	23.8	10.76–58.12	< 0.001
Unspecific	12.18	2.79–87.07	0.003
Laterality			
Unspecified	Baseline	Baseline	Baseline
Central	0.2	0.05–0.82	0.03
Left	0.46	0.23–0.91	0.03
Right anterior left	0.13	0.06–0.3	< 0.001
Right	0.32	0.16–0.64	0.0015
Length	1.93	1.49–2.52	< 0.001
Duration	0.68	0.62–0.74	< 0.001

Age was significantly associated with tumor severity, with older patients more likely to have malignant tumors (OR = 1.61; 95% CI = 1.41–1.84; *p* < 0.001), while sex was not (OR = 1.1; 95% CI = 0.73–1.66; *p* = 0.66). Tumor site strongly influenced malignancy risk. Compared to mandibular tumors, those in the extraoral region were 108 times more likely to be malignant (OR = 108.02; *p* < 0.001), while tumors in the oral mucosa and salivary glands (OR = 23.8; *p* < 0.001), midface (OR = 3.88; *p* < 0.001), and unspecified sites (OR = 12.18; *p* = 0.003) also showed higher malignancy odds. Laterality was also significantly associated with tumor severity. Central (OR = 0.2; *p* = 0.03), left (OR = 0.46; *p* = 0.03), right (OR = 0.32; *p* = 0.0015), and right-anterior-left (RAL) tumors (OR = 0.13; *p* < 0.001) were less likely to be malignant than those with unspecified laterality. Greater tumor length, measured in centimeters at the time of presentation, was significantly associated with malignancy (OR = 2.11; *p* < 0.001), whereas duration before presenting to the clinic was inversely correlated, with shorter durations correlating with a higher malignancy risk (OR = 0.68; *p* < 0.001).

The predictive model’s performance in distinguishing malignant from benign tumors was evaluated using two validation approaches: data splitting and LOOCV.

**Fig. 3:**
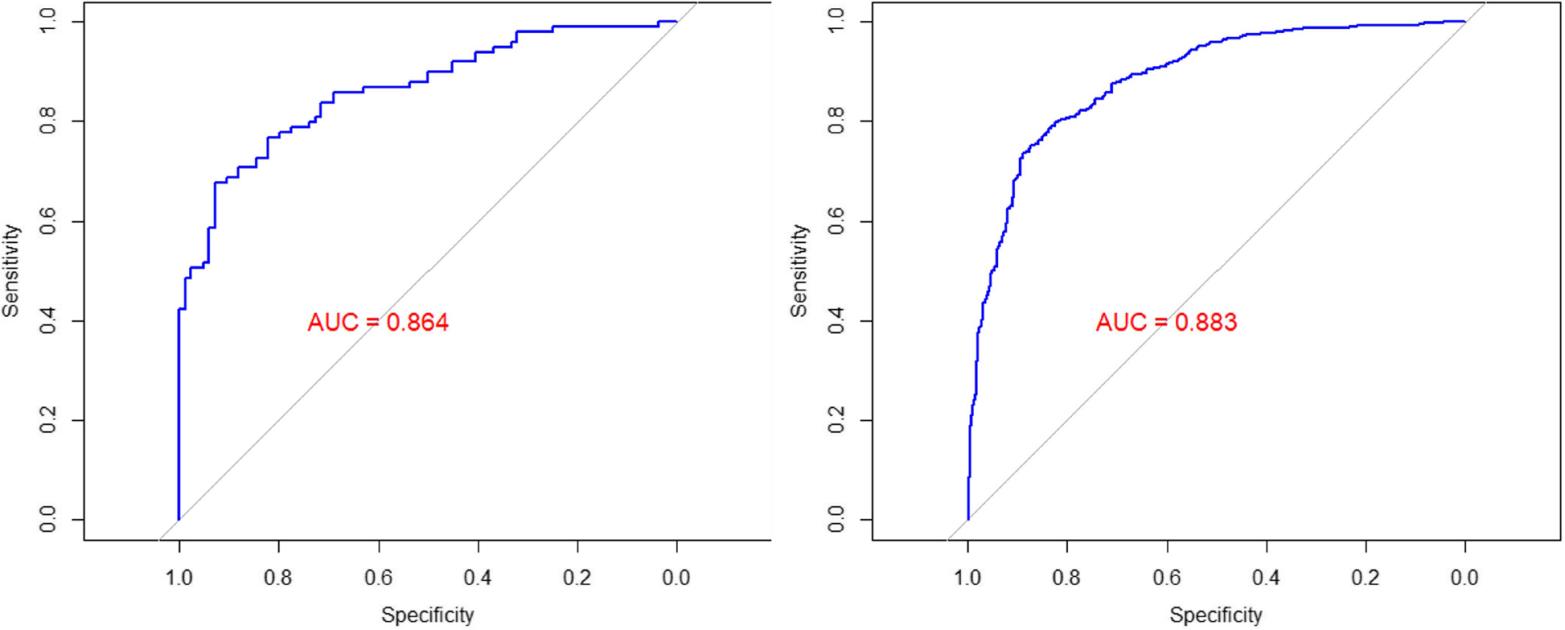
Receiver Operating Characteristic (ROC) Curves for Predictive Model Performance. Left panel: Data-splitting approach. Right panel: Leave-One-Out Cross-Validation (LOOCV) approach

For data splitting, the final logistic model achieved an AUC of 0.864 (Fig. 3):

$$\begin{aligned}&\log({\mathrm p}_{\mathrm i}/1-{\mathrm p}_{\mathrm i})=\log(0.1561)+\log(1.6693)\\&\ast\surd{\mathrm{Age}}_{\mathrm i}+\log(66.4797)\\&\ast\mathrm I({\mathrm{Site}}_{\mathrm i}=\mathrm{extraoral})+\log(3.6127)\\&\ast\mathrm I({\mathrm{Site}}_{\mathrm i}=\mathrm{midface})+\log(18.0803)\\&\ast\mathrm I({\mathrm{Site}}_{\mathrm i}=\mathrm{oral}\;\mathrm{mucosa}\;\mathrm{and}\;\mathrm{salivary}\;\mathrm{gland})\\&+\log(4.4657)\\&\ast\mathrm I({\mathrm{Site}}_{\mathrm i}=\mathrm{unspecified})+\log(0.2535)\\&\ast\mathrm I({\mathrm{Lat}}_{\mathrm i}=\mathrm{central})+\log(0.7860)\\&\ast\mathrm I({\mathrm{Lat}}_{\mathrm i}=\mathrm{left})+\log(0.3782)\\&\ast\mathrm I({\mathrm{Lat}}_{\mathrm i}=\mathrm{RAL})+\log(0.4279)\\&\ast\mathrm I({\mathrm{Lat}}_{\mathrm i}=\mathrm{right})+\log(0.6413)\ast\surd{\mathrm{Duration}}_{\mathrm i}\end{aligned}$$

For LOOCV, the model showed similar properties but with a slightly higher AUC of 0.883 (Fig. 3), indicating strong stability. Sensitivity and specificity analyses further assessed classification performance, revealing moderate sensitivity (0.727) for detecting malignancy and high specificity (0.833) for identifying benign tumors.

### Trend

**Fig. 4:**
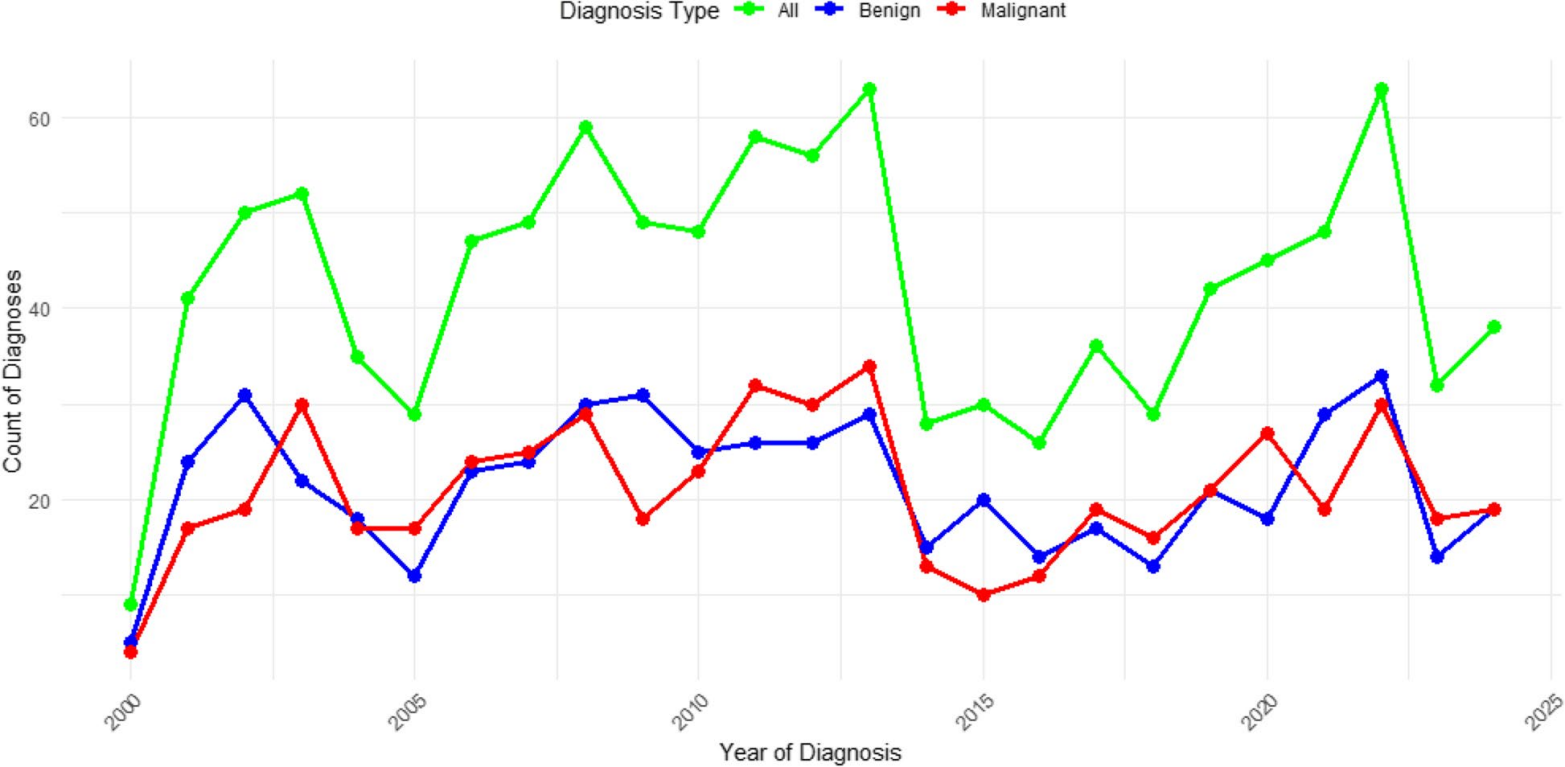
Trends in Tumor Diagnoses Over Time. Count of benign, malignant, and total tumor diagnoses by year

Overall, there was an upward trend in the number of tumors diagnosed over the years, with a notable decline observed between 2014 and 2021. In the earlier years, malignant cases outnumbered benign cases; however, over the past decade, benign cases have generally been more prevalent than malignant cases (Fig. 4).

## Discussion

The results of the current study provide important insights into patient demographics, tumor characteristics, and the performance of the predictive model.

### Univariate

Our study in South-Western Nigeria found a higher prevalence of orofacial tumors in males, consistent with reports from North-Western and North-Eastern Nigeria [25, 26]. However, a multicenter study on Nigerian children and research from the South-South region reported higher prevalence in females [9, 24]. Benign tumors were more than malignant cases and accounted for 50.75% of cases, aligning with findings from North-Western and South-South Nigeria [12, 24]. A similar proportion (51%) was reported in another South-Western study on adolescents and children [6]. While a multicenter study in Nigeria found an 85.4% prevalence in younger populations [9] which is consistent with pediatric studies in Ghana (69.66%) [10] and the Gambia, where benign tumors were even more common [29]. In contrast, studies from Uganda and England reported a predominance of malignant tumors [11, 19], likely due to differences in social, cultural, occupational, and climatic factors. Consistent with studies in other parts of Nigeria, other African countries, and Asia [11, 24, 25, 30–32], conventional ameloblastoma was the most common orofacial tumor and the most frequent benign tumor in this region. The mandible was the predominant site for ameloblastoma, with 87.78% (*n* = 237) of cases occurring in this location. This agrees with previous reports where ameloblastoma is reported to have a predilection for the mandible, reinforcing the well-documented predilection of this tumor for the lower jaw [33–35]. SCC was the most frequent malignancy which is in line with findings of previous Nigerian studies [24, 27], though some reports identified Burkitt’s lymphoma as the most common, particularly in adolescents [6, 25]. Similarly, pediatric studies in South Africa also found Burkitt’s lymphoma to be the leading malignancy [36]. Among SCC cases, the well-differentiated subtype was the most prevalent, consistent with findings from North-Eastern Nigeria [26].

### Bivariate

Age was significantly associated with tumor severity, with older patients more likely to have malignant tumors, aligning with previous studies [37]. This trend even in malignancies outside the orofacial region may be due to cumulative genetic mutations, prolonged carcinogen exposure, and age-related immune changes [38, 39]. Males also had a higher proportion of malignant tumors, consistent with prior reports [6, 9, 11, 27]. This observation may be attributed to several factors including lifestyle and occupational exposures, as men are more likely to engage in behaviors associated with increased cancer risk, such as tobacco smoking, alcohol consumption, and occupational exposure to carcinogens [40]. Tumor site was strongly associated with malignancy (*p* < 0.001), with the highest malignancy rates observed in the extraoral region (97.73%). This finding is clinically significant, as it may suggest a higher likelihood of aggressive tumor behavior, possibly due to these sites serving as common locations for metastasis. Laterality was also significant (*p* < 0.001), with unspecified laterality showing the highest malignancy rate (73.33%), which may suggest that these cases were diagnosed at an advanced stage when precise localization was more difficult due to extensive tissue involvement.

Interestingly, tumor size which was measured by length, breadth, and height was not associated with malignancy in this present study. This finding suggests that tumor dimensions alone may not be a reliable predictor of tumor severity as seen in other extraoral malignancies [41, 42], emphasizing the need to consider other clinical and histopathological factors when assessing tumor behavior. Additionally, shorter duration of symptoms before presentation was linked to higher odds of malignancy (*p* < 0.001), contradicting a previous study that found no association [24]. Age showed a negative correlation with the duration of symptoms (Spearman’s correlation = −0.112, *p* = 0.00067), indicating that older patients sought medical attention sooner after the onset of symptoms. This may be due to the higher prevalence of malignancy in older individuals, as observed in this current study. Malignant tumors often progress rapidly and present with alarming symptoms such as pain, ulceration, or rapid growth, which likely prompt earlier medical consultation compared to benign lesions [43]. Tumor site however was not associated with sex (*p* = 0.054).

### Multivariate

The multivariable analysis confirmed key associations. Age remained a strong predictor, with each additional year increasing malignancy risk by 61%, reinforcing the link between aging and cancer [37]. Tumor location was highly predictive of tumor severity where extraoral tumors were 108 times more likely to be malignant than mandibular tumors, possibly due to metastasis at diagnosis [44]. Laterality also played a role; centrally located tumors were less likely to be malignant than those with unspecified laterality, suggesting that tumors without clear laterality may be more aggressive. The duration of symptoms was inversely related to malignancy (OR = 0.68, 95% CI: 0.62–0.74, *p* < 0.001), indicating that malignant tumors progress more rapidly, leading to early onset of symptoms and faster clinical presentation [43].

Our predictive model that included age, sex, site, laterality and duration demonstrated considerable performance, achieving an AUC of 0.864 using the data-splitting approach and 0.883 with LOOCV. These high AUC values indicate the model’s considerable discriminative ability in distinguishing between benign and malignant tumors, suggesting its potential as a valuable tool for clinical decision-making [45]. Further evaluation of sensitivity and specificity confirmed the model’s diagnostic performance, with sensitivity and specificity values of 0.727 and 0.833; respectively. While the model demonstrates high specificity, making it effective at identifying benign tumors and reducing the risk of unnecessary interventions, its moderate sensitivity suggests room for improvement in correctly identifying malignant cases [46]. Improving the model sensitivity could have important implications for early malignancy detection. However, further validation with independent datasets is essential to assess the model’s generalizability and clinical applicability.

For clinical utility, we developed a simple Excel-based risk calculator derived from the multivariable model. Clinicians can input a patient’s age, tumor site, laterality, and symptom duration. The calculator automatically computes the predicted probability of malignancy and assigns a risk category: Low (probability < 0.3), Moderate (0.3 ≤ probability < 0.7), or High (probability ≥ 0.7). For example, a 40-year-old patient presenting with a 12-month history of a mandibular tumor of unspecified laterality would receive a predicted probability of 0.46, corresponding to a suggested moderate risk classification. This tool translates statistical modeling into an actionable format that can guide clinical decisions, especially in resource-limited settings where access to advanced imaging or pathology services may be delayed. It can help prioritize biopsy and referral decisions, ensuring that patients with the highest risk receive expedited evaluation. Future work should focus on prospective validation and refinement of thresholds to optimize sensitivity and specificity across different clinical contexts.

This current study showed an overall increase in tumor diagnoses, with a decline between 2014–2021, followed by a sharp rise in 2022 and another decline. This pattern mirrors trends observed in a pediatric study [9]. The 2022 surge likely resulted from patients seeking evaluations after the COVID-19 lockdown, a trend that later stabilized. Initially, malignant tumors were more common, but over the past decade, benign tumors have outnumbered malignant ones, suggesting possible shifts in presentation patterns or improvements in early detection.

### Limitations

Our study has some limitations. Firstly, this study was conducted at a single tertiary institution, which may limit the generalizability of the findings to broader populations in Nigeria and other regions. Second, while consolidating tumor locations into broader categories improved model performance, it may have overlooked important anatomical distinctions and site-specific malignancy risks, potentially reducing the precision of clinical interpretations. Third, although the predictive model demonstrated considerate performance, external validation with independent datasets is necessary to evaluate its applicability in different clinical settings. In addition, important factors such as genetic predisposition, environmental exposures, and lifestyle behaviors including tobacco use, alcohol consumption, viral infections like human papillomavirus, and family history were not available in the dataset. These are well established contributors to head and neck cancer risk, and their absence may limit the model’s ability to fully explain variation in tumor severity. This study also did not account for tumors that were recurrent, secondary, or metastatic, as such details were not consistently recorded. The tissue of origin for each tumor was not reported. Future studies should aim to include these variables to build a more complete and useful model for clinical care.

Although we provide an Excel-based calculator to illustrate how our model could be applied in practice, this tool has not undergone external validation. Its primary purpose is to demonstrate the framework and potential utility of our model in risk estimation.

## Conclusions

This study provides valuable insights into the demographic and clinical factors associated with tumor malignancy, reinforcing the role of age, sex, tumor site, and symptom duration in malignancy risk. Our predictive model demonstrated considerate diagnostic performance, suggesting its potential for aiding clinical decision-making in distinguishing between benign and malignant tumors. Future work should focus on refining the model’s sensitivity and evaluating its external validity in different settings.

## Supplementary Information

Supplementary Material 1.

## Data Availability

The data that support the findings of this study are available upon request. The data are not publicly available due to privacy or ethical restrictions.

## Abbreviations

SCC: Squamous cell carcinoma
IQR: Interquartile range
rs: Spearman’s rank correlation coefficient
GVIF: Generalized variance inflation factor
LOOCV: Leave-one-out cross-validation
AIC: Akaike information criterion
ROC: Receiver operating curve
AUC: Area under the curve
OR: Odds ratio
